# *N*,*N*′-Bis­[(*E*)-2-fluoro­benzyl­idene]-1-(2-fluoro­phen­yl)methane­diamine

**DOI:** 10.1107/S1600536810001984

**Published:** 2010-01-23

**Authors:** Jerry P. Jasinski, Ray J. Butcher, Q. N. M. Hakim Al-Arique, H. S. Yathirajan, B. Narayana

**Affiliations:** a Department of Chemistry, Keene State College, 229 Main Street, Keene, NH 03435-2001, USA; b Department of Chemistry, Howard University, 525 College Street NW, Washington, DC 20059, USA; c Department of Studies in Chemistry, University of Mysore, Manasagangotri, Mysore 570 006, India; d Department of Studies in Chemistry, Mangalore University, Mangalagangotri 574 199, India

## Abstract

In the title compound, C_21_H_15_F_3_N_2_, the benzene ring bonded to the central C atom forms dihedral angles of 77.5 (7) and 89.0 (5)°, respectively, with the remaining two benzene rings. Weak inter­molecular C—H⋯F hydrogen bonds link the mol­ecules into chains propagated in [101]. The crystal packing exhibits weak π–π inter­actions as evidenced by relatively short distances between the centroids of the aromatic rings [3.820 (7) and 3.971 (5) Å]. A MOPAC PM3 optimization of the mol­ecular geometry *in vacuo* supports a suggestion that inter­molecular forces have a significnt influence on the mol­ecular conformation in the crystal.

## Related literature

For aromatic aldehyde reactions, see Williams & Bailar (1959 ▶). For kinetics of hydro­benzamides, see Crampton *et al.* (1997 ▶). For conventional preparation of hydro­benzamides, see Kamal & Qureshi (1963 ▶). For related structures, see: Corey & Kuhnle (1997 ▶); Karupaiyan *et al.* (1998 ▶); Saigo *et al.* (1986 ▶). For bond-length data, see: Allen *et al.* (1987 ▶). For the synthesis of nitro­gen-containing heterocyclic compounds, see Kupfer & Brinker (1996 ▶). For MOPAC PM3 calculations, see Schmidt & Polik (2007 ▶). 
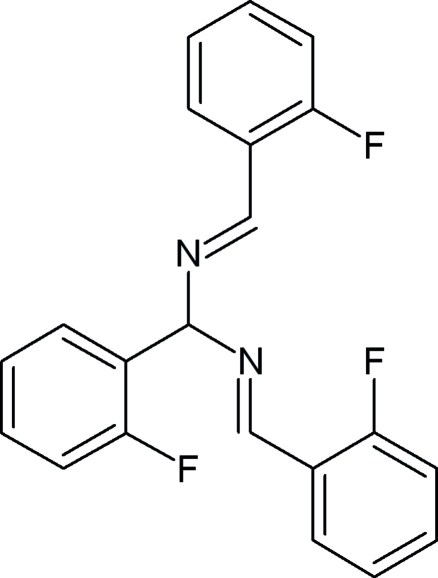


## Experimental

### 

#### Crystal data

C_21_H_15_F_3_N_2_
*M* *_r_* = 352.35
Triclinic, 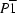

*a* = 8.0215 (5) Å
*b* = 9.3740 (4) Å
*c* = 11.9744 (6) Å
α = 99.184 (4)°
β = 93.179 (5)°
γ = 108.165 (5)°
*V* = 839.23 (8) Å^3^
*Z* = 2
Mo *K*α radiation
μ = 0.11 mm^−1^
*T* = 200 K
0.49 × 0.29 × 0.22 mm

#### Data collection

Oxford Diffraction Gemini diffractometer
11550 measured reflections
5484 independent reflections
3292 reflections with *I* > 2σ(*I*)
*R*_int_ = 0.025

#### Refinement

*R*[*F* ^2^ > 2σ(*F* ^2^)] = 0.052
*wR*(*F* ^2^) = 0.152
*S* = 1.00
5484 reflections
235 parameters
H-atom parameters constrained
Δρ_max_ = 0.57 e Å^−3^
Δρ_min_ = −0.20 e Å^−3^

### 

Data collection: *CrysAlis PRO* (Oxford Diffraction, 2007 ▶); cell refinement: *CrysAlis PRO*; data reduction: *CrysAlis PRO*; program(s) used to solve structure: *SHELXS97* (Sheldrick, 2008 ▶); program(s) used to refine structure: *SHELXL97* (Sheldrick, 2008 ▶); molecular graphics: *SHELXTL* (Sheldrick, 2008 ▶); software used to prepare material for publication: *SHELXTL*.

## Supplementary Material

Crystal structure: contains datablocks global, I. DOI: 10.1107/S1600536810001984/cv2683sup1.cif

Structure factors: contains datablocks I. DOI: 10.1107/S1600536810001984/cv2683Isup2.hkl

Additional supplementary materials:  crystallographic information; 3D view; checkCIF report

## Figures and Tables

**Table 1:** Hydrogen-bond geometry (Å, °)

*D*—H⋯*A*	*D*—H	H⋯*A*	*D*⋯*A*	*D*—H⋯*A*
C5*B*—H5*BA*⋯F1*A*^i^	0.95	2.53	3.3871 (16)	151

Symmetry code: (i) 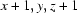.

**Fig. 1.**
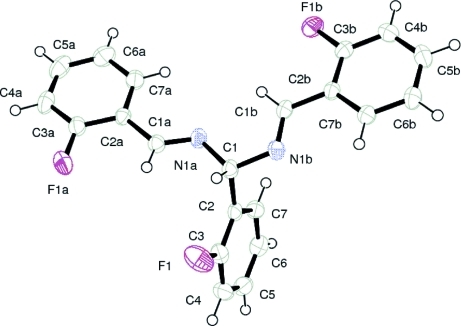
Molecular structure of (I), C21H15F3N2, showing the atom labeling scheme and 50% probability displacement ellipsoids.

**Fig. 2.**
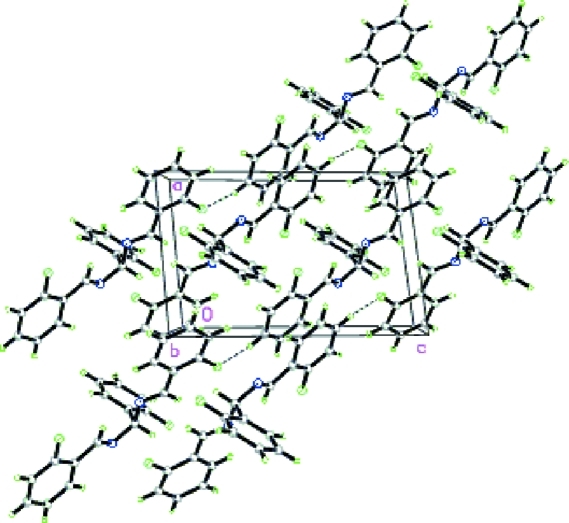
The molecular packing for (I) viewed down the b axis. Dashed lines indicate weak C—H···F intermolecular hydrogen bond interactions which link the molecule into chains propagating obliquely along the c axis.

**Fig. 3.**
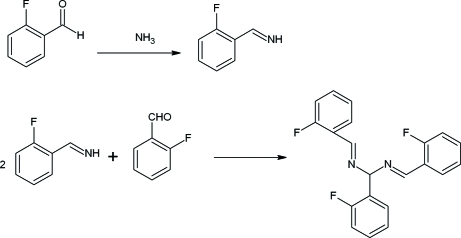
Synthetic scheme for C21H15F3N2, (I).

## supplementary crystallographic information

### Comment

Reaction of aromatic aldehydes with ammonia leads to the long-known compounds called "hydrobenzamides" (Williams & Bailar, 1959). Owing to their unique structural features and reactivity, these compounds have been recognized as potential key intermediates for the synthesis of a variety of nitrogen containing heterocyclic compounds (Kupfer & Brinker, 1996). Extensive studies on kinetics and mechanism of formation of hydrobenzamides from aromatic aldehydes and ammonia have been well documented (Crampton *et al.* 1997). The only conventional method available for the preparation of these compounds involves the reaction of aldehydes with ammonia, a complex reversible reaction which takes days to weeks for completion (Kamal & Qureshi, 1963). Moreover, protic solvents used in this reaction such as methanol or water enhance the reversible conversion of products into starting aldehydes, thereby reducing the yields even after longer reaction times. Due to the importance of these compounds, we report the crystal structure of a newly synthesized derivative, C_21_H_15_F_3_N_2_, (I).

The title compound, C_21_H_15_F_3_N_2_, (I), consists of a 2-fluorophenyl group and a *N*,*N*'-bis[(*E*)-(2-fluorophenyl)methylidene]methanediamine group bonded to a methane carbon, C1 (Fig. 1). The benzene ring bonded to the central methyl carbon atom forms dihedral angles of 77.5 (7)° and 89.0 (5)°, respectively, with the remaining two benzene rings. The dihedral angle between the mean planes of the remaining two benzene rings is 15.7 (7)°. Five of the angles around the methane carbon, C1, are in the vicinity of the 108°-109° range (N1A—C1—C2; 109.45 (11)°, N1B—C1—C2; 108.04 (10)°, C2—C1—H1A; 108.(2)°, N1A—C1—H1A; 108.(2)°, N1B—C1—H1A; 108.(2)°) with only the N1A—C1—N2A angle measuring 114.48 (10)° giving it a slightly distorted *sp*^3^ configuration in the direction of the two nitrogen atoms. Bond lengths and bond angles are all within expected ranges (Allen *et al.*, 1987).

Crystal packing is influenced by weak C—H···F intermolecular hydrogen bond interactions which link the molecule into chains propagating obliquely along the *c* axis in the direction [101] (Fig. 2). In addition, weak *Cg*2···*Cg*2 (3.971 (5) Å; -*x*, 1 - *y*, -*z*) and *Cg*3···*Cg*3 (3.820 (7) Å; 2 - *x*, 2 - *y*, 1 - *z*) π-π intermolecular interactions are observed with slippage distances of 1.81 (4) Å and 1.76 (5) Å, respectively. (*Cg*2, *Cg*3 = ring centroids for C2A—C7A and C2B—C7B, respectively).

In support of these observations, a MOPAC PM3 calculation was performed on the C_21_H_15_F_3_N_2_, molecule with WebMO Pro (Schmidt & Polik, 2007) (PM3, Parameterized Model 3) approximation together with the Hartree-Fock closed-shell (restricted) wavefunction was used and minimizations were teminnated at an r.m.s. gradient of less than 0.01 kJ mol^-1^ Å^-1^.). While the bond distances did not appear to change significantly, selected bond and torsion angles were noticeably different. The bond angle for N1A—C1A—N1B (114.48 (10)° *versus* 111.3°) is shorter and for C2A—C3A—F1A (117.81 (12)° *versus* 120.4°) is wider after the calculation. The torsion angles for C1A—N1A—C1—C2 (86.45 (14)° *versus* 78.17°) and C1B—N1B—C1—C2 (124.39 (13)° *versus* 96.35°) are both much lower after the calculation indicating a much greater twist causing the two benzene rings to be further apart. This is supported by the PM3 calculated value of 36.79° (*versus*. 15.7 (7)° before the calculation) for the angle between the mean planes of the two benzene rings. In addition the angles between the mean planes of the two benzene rings with the C1 bonded benzene are 70.22° (*versus*.77.5 (7)°) and 82.32° (*versus*. 89.0 (5)°), respectively, after the calculation. This suggests that small changes in some bond distances and selectively in some bond and torsion angles, especially involving the diamine nitrogen atoms have been infuenced by the collective effect of all of the weak intermolecular interactions that have been observed in the crystal packing.

### Experimental

10 ml of 25% methanolic ammonia was added to a solution of 2 g of 2-flurobenzaldehyde in 10 ml me thanol and left to stand at ambient temperature for 2 days, during which the crystalline products separated out (Fig. 3). The crude crystals were filtered off, washed with cold methanol. Good quality *x*-ray grade crystals were obtained by the slow evaporation of the solution in ethyl acetate (m.p.: 425–427 K). Analysis for the title compound C_21_H_15_F_3_N_2_: Found (calculated): C: 71.75 (71.82); H: 4.26 (4.29); N: 7.90 (7.95).

### Refinement

All of the H atoms were placed in their calculated positions and then refined using the riding model with C—H = 0.95 Å, and with *U*_iso_(H) = 1.18–1.20*U*_eq_(C).

### Crystal data

**Table:**

C_21_H_15_F_3_N_2_	*Z* = 2
*M**_r_* = 352.35	*F*(000) = 364
Triclinic, *P*1	*D*_x_ = 1.394 Mg m^−^^3^
Hall symbol: -P 1	Mo *K*α radiation, λ = 0.71073 Å
*a* = 8.0215 (5) Å	Cell parameters from 4026 reflections
*b* = 9.3740 (4) Å	θ = 4.6–32.4°
*c* = 11.9744 (6) Å	µ = 0.11 mm^−^^1^
α = 99.184 (4)°	*T* = 200 K
β = 93.179 (5)°	Prism, colourless
γ = 108.165 (5)°	0.49 × 0.29 × 0.22 mm
*V* = 839.23 (8) Å^3^	

### Data collection

**Table:**

Oxford Diffraction Gemini diffractometer	3292 reflections with *I* > 2σ(*I*)
Radiation source: fine-focus sealed tube	*R*_int_ = 0.025
graphite	θ_max_ = 32.5°, θ_min_ = 4.6°
Detector resolution: 10.5081 pixels mm^-1^	*h* = −11→12
φ and ω scans	*k* = −14→13
11550 measured reflections	*l* = −16→17
5484 independent reflections	

### Refinement

**Table:**

Refinement on *F*^2^	Primary atom site location: structure-invariant direct methods
Least-squares matrix: full	Secondary atom site location: difference Fourier map
*R*[*F*^2^ > 2σ(*F*^2^)] = 0.052	Hydrogen site location: inferred from neighbouring sites
*wR*(*F*^2^) = 0.152	H-atom parameters constrained
*S* = 1.00	*w* = 1/[σ^2^(*F*_o_^2^) + (0.0841*P*)^2^] where *P* = (*F*_o_^2^ + 2*F*_c_^2^)/3
5484 reflections	(Δ/σ)_max_ < 0.001
235 parameters	Δρ_max_ = 0.57 e Å^−^^3^
0 restraints	Δρ_min_ = −0.20 e Å^−^^3^

### Special details

**Table:**

Geometry. All e.s.d.'s (except the e.s.d. in the dihedral angle between two l.s. planes) are estimated using the full covariance matrix. The cell e.s.d.'s are taken into account individually in the estimation of e.s.d.'s in distances, angles and torsion angles; correlations between e.s.d.'s in cell parameters are only used when they are defined by crystal symmetry. An approximate (isotropic) treatment of cell e.s.d.'s is used for estimating e.s.d.'s involving l.s. planes.
Refinement. Refinement of *F*^2^ against ALL reflections. The weighted *R*-factor *wR* and goodness of fit *S* are based on *F*^2^, conventional *R*-factors *R* are based on *F*, with *F* set to zero for negative *F*^2^. The threshold expression of *F*^2^ > σ(*F*^2^) is used only for calculating *R*-factors(gt) *etc*. and is not relevant to the choice of reflections for refinement. *R*-factors based on *F*^2^ are statistically about twice as large as those based on *F*, and *R*- factors based on ALL data will be even larger.

### Fractional atomic coordinates and isotropic or equivalent isotropic displacement parameters (Å^2^)

**Table:**

	*x*	*y*	*z*	*U*_iso_*/*U*_eq_	
F1	0.62912 (14)	1.01485 (11)	0.09363 (8)	0.0596 (3)	
F1A	0.21867 (14)	0.54600 (10)	−0.14842 (7)	0.0511 (3)	
F1B	0.62463 (12)	0.41513 (8)	0.48075 (7)	0.0434 (2)	
N1A	0.43939 (15)	0.62936 (12)	0.17778 (9)	0.0315 (3)	
N1B	0.70316 (15)	0.77388 (12)	0.31351 (9)	0.0314 (3)	
C1	0.58583 (18)	0.77356 (14)	0.21571 (11)	0.0297 (3)	
H1A	0.6576	0.7941	0.1509	0.036*	
C2	0.51189 (17)	0.90345 (13)	0.24663 (10)	0.0282 (3)	
C3	0.53477 (19)	1.01805 (15)	0.18400 (11)	0.0344 (3)	
C4	0.4682 (2)	1.13682 (16)	0.20930 (13)	0.0424 (4)	
H4A	0.4871	1.2137	0.1640	0.051*	
C5	0.3733 (2)	1.14203 (16)	0.30200 (13)	0.0427 (4)	
H5A	0.3249	1.2222	0.3203	0.051*	
C6	0.3491 (2)	1.03019 (16)	0.36807 (12)	0.0404 (3)	
H6A	0.2855	1.0345	0.4324	0.049*	
C7	0.4174 (2)	0.91224 (15)	0.34048 (11)	0.0348 (3)	
H7A	0.3997	0.8359	0.3862	0.042*	
C1A	0.38045 (18)	0.59817 (14)	0.07361 (10)	0.0284 (3)	
H1AA	0.4326	0.6673	0.0256	0.034*	
C2A	0.23439 (17)	0.45892 (13)	0.02440 (10)	0.0269 (3)	
C3A	0.15517 (19)	0.43569 (15)	−0.08628 (11)	0.0325 (3)	
C4A	0.0176 (2)	0.30824 (17)	−0.13529 (12)	0.0411 (4)	
H4AA	−0.0330	0.2975	−0.2111	0.049*	
C5A	−0.0459 (2)	0.19599 (17)	−0.07250 (14)	0.0479 (4)	
H5AA	−0.1414	0.1067	−0.1050	0.057*	
C6A	0.0291 (2)	0.21259 (16)	0.03835 (13)	0.0468 (4)	
H6AA	−0.0148	0.1346	0.0813	0.056*	
C7A	0.1674 (2)	0.34260 (15)	0.08573 (11)	0.0361 (3)	
H7AA	0.2180	0.3532	0.1615	0.043*	
C1B	0.67867 (17)	0.65547 (14)	0.35664 (10)	0.0281 (3)	
H1BA	0.5831	0.5652	0.3263	0.034*	
C2B	0.80098 (17)	0.65923 (13)	0.45521 (10)	0.0276 (3)	
C3B	0.76856 (18)	0.54090 (14)	0.51567 (11)	0.0305 (3)	
C4B	0.8769 (2)	0.54521 (16)	0.61103 (12)	0.0367 (3)	
H4BA	0.8495	0.4630	0.6515	0.044*	
C5B	1.0254 (2)	0.67148 (17)	0.64619 (12)	0.0409 (4)	
H5BA	1.1014	0.6764	0.7115	0.049*	
C6B	1.0645 (2)	0.79119 (16)	0.58684 (12)	0.0408 (4)	
H6BA	1.1675	0.8775	0.6110	0.049*	
C7B	0.95321 (19)	0.78464 (14)	0.49220 (11)	0.0335 (3)	
H7BA	0.9809	0.8669	0.4518	0.040*	

### Atomic displacement parameters (Å^2^)

**Table:**

	*U*^11^	*U*^22^	*U*^33^	*U*^12^	*U*^13^	*U*^23^
F1	0.0633 (7)	0.0747 (7)	0.0551 (6)	0.0276 (6)	0.0245 (5)	0.0366 (5)
F1A	0.0650 (7)	0.0515 (5)	0.0334 (4)	0.0138 (5)	−0.0069 (4)	0.0125 (4)
F1B	0.0381 (5)	0.0310 (4)	0.0537 (5)	0.0007 (4)	0.0004 (4)	0.0093 (4)
N1A	0.0280 (6)	0.0319 (5)	0.0289 (5)	0.0041 (5)	−0.0036 (5)	0.0032 (4)
N1B	0.0261 (6)	0.0322 (5)	0.0328 (5)	0.0068 (5)	−0.0042 (5)	0.0047 (4)
C1	0.0253 (7)	0.0308 (6)	0.0286 (6)	0.0035 (5)	−0.0018 (5)	0.0056 (5)
C2	0.0223 (6)	0.0282 (6)	0.0279 (6)	0.0011 (5)	−0.0053 (5)	0.0043 (5)
C3	0.0288 (7)	0.0393 (7)	0.0314 (6)	0.0039 (6)	0.0012 (6)	0.0117 (6)
C4	0.0439 (9)	0.0345 (7)	0.0473 (8)	0.0078 (7)	−0.0039 (7)	0.0168 (6)
C5	0.0429 (9)	0.0327 (7)	0.0489 (8)	0.0125 (6)	−0.0068 (7)	0.0008 (6)
C6	0.0397 (9)	0.0411 (7)	0.0363 (7)	0.0101 (7)	0.0041 (6)	0.0016 (6)
C7	0.0379 (8)	0.0317 (6)	0.0317 (6)	0.0062 (6)	0.0030 (6)	0.0081 (5)
C1A	0.0271 (7)	0.0291 (6)	0.0282 (6)	0.0082 (5)	0.0020 (5)	0.0056 (5)
C2A	0.0252 (7)	0.0280 (6)	0.0266 (6)	0.0106 (5)	0.0001 (5)	−0.0001 (5)
C3A	0.0327 (8)	0.0361 (7)	0.0294 (6)	0.0146 (6)	−0.0004 (6)	0.0023 (5)
C4A	0.0330 (8)	0.0471 (8)	0.0371 (7)	0.0152 (7)	−0.0087 (6)	−0.0097 (6)
C5A	0.0316 (8)	0.0408 (8)	0.0581 (10)	0.0038 (7)	−0.0024 (7)	−0.0103 (7)
C6A	0.0441 (10)	0.0343 (7)	0.0544 (9)	0.0031 (7)	0.0059 (8)	0.0062 (7)
C7A	0.0372 (8)	0.0360 (7)	0.0326 (7)	0.0095 (6)	0.0015 (6)	0.0051 (5)
C1B	0.0236 (7)	0.0283 (6)	0.0282 (6)	0.0052 (5)	0.0004 (5)	0.0002 (5)
C2B	0.0249 (7)	0.0278 (6)	0.0277 (6)	0.0080 (5)	−0.0003 (5)	0.0008 (5)
C3B	0.0278 (7)	0.0264 (6)	0.0345 (6)	0.0066 (5)	0.0037 (6)	0.0022 (5)
C4B	0.0420 (9)	0.0373 (7)	0.0360 (7)	0.0171 (7)	0.0054 (6)	0.0126 (6)
C5B	0.0406 (9)	0.0509 (8)	0.0327 (7)	0.0182 (7)	−0.0042 (6)	0.0077 (6)
C6B	0.0325 (8)	0.0401 (7)	0.0413 (7)	0.0041 (6)	−0.0087 (6)	0.0033 (6)
C7B	0.0306 (7)	0.0295 (6)	0.0368 (7)	0.0055 (6)	−0.0025 (6)	0.0064 (5)

### Geometric parameters (Å, °)

**Table:**

F1—C3	1.3562 (16)	C2A—C7A	1.3968 (18)
F1A—C3A	1.3571 (16)	C3A—C4A	1.3681 (19)
F1B—C3B	1.3558 (15)	C4A—C5A	1.376 (2)
N1A—C1A	1.2637 (15)	C4A—H4AA	0.9500
N1A—C1	1.4725 (16)	C5A—C6A	1.391 (2)
N1B—C1B	1.2632 (15)	C5A—H5AA	0.9500
N1B—C1	1.4602 (16)	C6A—C7A	1.380 (2)
C1—C2	1.5173 (18)	C6A—H6AA	0.9500
C1—H1A	1.0000	C7A—H7AA	0.9500
C2—C3	1.3782 (18)	C1B—C2B	1.4811 (17)
C2—C7	1.3958 (19)	C1B—H1BA	0.9500
C3—C4	1.377 (2)	C2B—C3B	1.3856 (17)
C4—C5	1.383 (2)	C2B—C7B	1.3957 (17)
C4—H4A	0.9500	C3B—C4B	1.3833 (18)
C5—C6	1.385 (2)	C4B—C5B	1.380 (2)
C5—H5A	0.9500	C4B—H4BA	0.9500
C6—C7	1.383 (2)	C5B—C6B	1.387 (2)
C6—H6A	0.9500	C5B—H5BA	0.9500
C7—H7A	0.9500	C6B—C7B	1.3846 (18)
C1A—C2A	1.4656 (17)	C6B—H6BA	0.9500
C1A—H1AA	0.9500	C7B—H7BA	0.9500
C2A—C3A	1.3914 (16)		
			
C1A—N1A—C1	116.40 (11)	C4A—C3A—C2A	123.58 (13)
C1B—N1B—C1	120.47 (10)	C3A—C4A—C5A	118.57 (13)
N1B—C1—N1A	114.48 (10)	C3A—C4A—H4AA	120.7
N1B—C1—C2	108.04 (10)	C5A—C4A—H4AA	120.7
N1A—C1—C2	109.45 (11)	C4A—C5A—C6A	120.35 (13)
N1B—C1—H1A	108.2	C4A—C5A—H5AA	119.8
N1A—C1—H1A	108.2	C6A—C5A—H5AA	119.8
C2—C1—H1A	108.2	C7A—C6A—C5A	119.82 (14)
C3—C2—C7	116.89 (12)	C7A—C6A—H6AA	120.1
C3—C2—C1	121.87 (12)	C5A—C6A—H6AA	120.1
C7—C2—C1	121.24 (11)	C6A—C7A—C2A	121.23 (12)
F1—C3—C4	118.24 (12)	C6A—C7A—H7AA	119.4
F1—C3—C2	118.47 (13)	C2A—C7A—H7AA	119.4
C4—C3—C2	123.29 (13)	N1B—C1B—C2B	119.11 (11)
C3—C4—C5	118.71 (13)	N1B—C1B—H1BA	120.4
C3—C4—H4A	120.6	C2B—C1B—H1BA	120.4
C5—C4—H4A	120.6	C3B—C2B—C7B	117.18 (11)
C4—C5—C6	119.87 (14)	C3B—C2B—C1B	121.92 (11)
C4—C5—H5A	120.1	C7B—C2B—C1B	120.89 (11)
C6—C5—H5A	120.1	F1B—C3B—C4B	118.11 (12)
C7—C6—C5	120.09 (14)	F1B—C3B—C2B	119.07 (11)
C7—C6—H6A	120.0	C4B—C3B—C2B	122.82 (12)
C5—C6—H6A	120.0	C5B—C4B—C3B	118.61 (13)
C6—C7—C2	121.14 (13)	C5B—C4B—H4BA	120.7
C6—C7—H7A	119.4	C3B—C4B—H4BA	120.7
C2—C7—H7A	119.4	C4B—C5B—C6B	120.43 (12)
N1A—C1A—C2A	122.22 (12)	C4B—C5B—H5BA	119.8
N1A—C1A—H1AA	118.9	C6B—C5B—H5BA	119.8
C2A—C1A—H1AA	118.9	C7B—C6B—C5B	119.84 (13)
C3A—C2A—C7A	116.45 (12)	C7B—C6B—H6BA	120.1
C3A—C2A—C1A	121.58 (11)	C5B—C6B—H6BA	120.1
C7A—C2A—C1A	121.97 (11)	C6B—C7B—C2B	121.11 (12)
F1A—C3A—C4A	118.62 (11)	C6B—C7B—H7BA	119.4
F1A—C3A—C2A	117.81 (12)	C2B—C7B—H7BA	119.4
			
C1B—N1B—C1—N1A	2.17 (18)	C7A—C2A—C3A—C4A	0.7 (2)
C1B—N1B—C1—C2	124.39 (13)	C1A—C2A—C3A—C4A	−179.39 (13)
C1A—N1A—C1—N1B	−152.10 (12)	F1A—C3A—C4A—C5A	179.56 (13)
C1A—N1A—C1—C2	86.45 (14)	C2A—C3A—C4A—C5A	−0.4 (2)
N1B—C1—C2—C3	122.02 (13)	C3A—C4A—C5A—C6A	−0.1 (2)
N1A—C1—C2—C3	−112.72 (13)	C4A—C5A—C6A—C7A	0.2 (2)
N1B—C1—C2—C7	−57.86 (15)	C5A—C6A—C7A—C2A	0.0 (2)
N1A—C1—C2—C7	67.40 (14)	C3A—C2A—C7A—C6A	−0.5 (2)
C7—C2—C3—F1	178.64 (11)	C1A—C2A—C7A—C6A	179.60 (14)
C1—C2—C3—F1	−1.25 (18)	C1—N1B—C1B—C2B	179.50 (12)
C7—C2—C3—C4	−0.6 (2)	N1B—C1B—C2B—C3B	171.37 (12)
C1—C2—C3—C4	179.50 (13)	N1B—C1B—C2B—C7B	−7.51 (19)
F1—C3—C4—C5	−179.35 (13)	C7B—C2B—C3B—F1B	−178.25 (12)
C2—C3—C4—C5	−0.1 (2)	C1B—C2B—C3B—F1B	2.83 (19)
C3—C4—C5—C6	0.9 (2)	C7B—C2B—C3B—C4B	1.9 (2)
C4—C5—C6—C7	−0.9 (2)	C1B—C2B—C3B—C4B	−177.00 (13)
C5—C6—C7—C2	0.2 (2)	F1B—C3B—C4B—C5B	178.88 (12)
C3—C2—C7—C6	0.54 (19)	C2B—C3B—C4B—C5B	−1.3 (2)
C1—C2—C7—C6	−179.57 (12)	C3B—C4B—C5B—C6B	0.0 (2)
C1—N1A—C1A—C2A	−179.66 (11)	C4B—C5B—C6B—C7B	0.5 (2)
N1A—C1A—C2A—C3A	171.24 (13)	C5B—C6B—C7B—C2B	0.2 (2)
N1A—C1A—C2A—C7A	−8.8 (2)	C3B—C2B—C7B—C6B	−1.3 (2)
C7A—C2A—C3A—F1A	−179.31 (12)	C1B—C2B—C7B—C6B	177.59 (13)
C1A—C2A—C3A—F1A	0.63 (19)		

### Hydrogen-bond geometry (Å, °)

**Table:**

*D*—H···*A*	*D*—H	H···*A*	*D*···*A*	*D*—H···*A*
C5B—H5BA···F1A^i^	0.95	2.53	3.3871 (16)	151

Symmetry codes: (i) *x*+1, *y*, *z*+1.
